# Six-Day Fasting Causes Temporary Increases in Both Antioxidant Capacity and Oxidative Stress in Healthy Young Men: A Randomized Controlled Trial

**DOI:** 10.3390/antiox14030269

**Published:** 2025-02-26

**Authors:** Marius Brazaitis, Katerina Židonienė, Nerijus Eimantas, Rima Solianik

**Affiliations:** Institute of Sport Science and Innovations, Lithuanian Sports University, 44221 Kaunas, Lithuania; marius.brazaitis@lsu.lt (M.B.); katerina.zidoniene@gmail.com (K.Ž.); nerijus.eimantas@lsu.lt (N.E.)

**Keywords:** males, malondialdehyde, oxidative status, starvation, total antioxidant capacity

## Abstract

The impact of prolonged fasting on human oxidative stress (OS) levels and antioxidant defence mechanisms remains poorly understood. The aim of this current study was to investigate the redox response to a 6-day fast in a cohort of healthy men. Twenty-six participants were randomly allocated to a 6-day complete fasting or a control trial. Sympathetic activity, substrate oxidation, redox status, blood glucose, ketones, and testosterone concentrations were assessed. Throughout the fasting period, ketone concentration and fat oxidation increased, and carbohydrate oxidation and glucose and testosterone concentrations decreased. Heart rate increased on fasting days 2 and 4 and returned to the pre-fasting level on fasting day 6. Malondialdehyde (MDA) concentration increased after fasting days 4 and 6, and this increase was accompanied by an increase in the total antioxidant capacity (TAC), but the TAC/MDA ratio remained constant. Notably, all fasting-evoked changes returned to the baseline values after resumption of the regular diet. Thus, prolonged fasting activated both antioxidant defence and OS, but the redox balance was maintained. Consistent with this response, ketone concentration and sympathetic nervous system activity increased, and testosterone concentration decreased. These variables returned to the pre-fasting state after resumption of the usual eating habits.

## 1. Introduction

Oxidative stress (OS) represents a potentially damaging imbalance of the redox states in the body involving excessive generation of reactive oxygen species and/or dysfunction of the protective antioxidant system [1]. OS contributes to the development of disorders such as rheumatoid arthritis [2], autoimmune liver diseases [3], cardiovascular diseases [4], and cancer [5]. OS also seems to play an important role in neurodegeneration by causing excitotoxicity, neuronal loss, and axonal damage, and is considered a key element in the onset and progression of several neurodegenerative diseases, including Parkinson’s disease, Alzheimer’s disease, multiple sclerosis, amyotrophic lateral sclerosis, and hereditary spastic paraplegia [6,7].

Intermittent and periodic fasting may help to prevent and treat diseases [8,9]. In contrast to the short and frequent fasting periods of intermittent fasting, which can be difficult to integrate into a daily routine, periodic fasting usually lasts between 2 and 7 days (2–3 days in mice and 4–7 days in humans) and is followed by a refeeding period of at least 1 week. As its name implies, periodic fasting can be periodic and does not require a specific interval but can be applied for one or several cycles as a preventive measure or in the treatment of a specific disease or condition [9].

To our knowledge, limited studies have evaluated the ability of prolonged zero-calorie fasting to modulate OS and total antioxidant system activity in humans. Five days of dry fasting, also called food and water deprivation, has been reported to increase the plasma total antioxidant capacity (TAC) [10]. Using an uncontrolled fasting duration of 3 to 11 days (average 7 days) with water provided ad libitum, one study found that fasting decreased the level of urinary malondialdehyde (MDA), a biomarker of OS [11]. Surprisingly, in that study, participants were allowed to perform exercise, including jogging and yoga, during the fasting programme [11], which may have affected OS [12,13]. It is unknown whether fasting duration can affect the redox status and, if so, whether any changes are temporary and appear only during the fasting period or are maintained for a longer time.

The metabolic switch from glucose to fatty acid–derived ketones typically occurs between 12 to 36 h after cessation of food consumption and allows the body to maintain optimal brain and body functioning [14]. However, experimental evidence suggests that ketones may act beyond their role as an energy substrate. Nazarewicz et al. [15] reported that a short-term (14 days) ketogenic diet with dietary restriction significantly improved total antioxidative status without causing OS in healthy young women. A later study observed that weight loss induced by 3 weeks of calorie restriction and exercise caused OS in teenagers and that a ketogenic diet could prevent this OS [16]. Other studies have indicated that, during prolonged fasting in healthy people, the production rate and concentration of ketones increase markedly during the early phase of fasting and reach a plateau after about 5 days [17,18,19], which suggests that the TAC may increase progressively during prolonged fasting. Stress hormones such as corticosteroids and catecholamines can modulate OS status [20]. Recent studies have reported that the morning cortisol concentration is not affected [19], but epinephrine concentration increases in the early phase of fasting but not after a prolonged 6-day fast [19,21]. Experimental studies using mostly animal models and cell cultures have shown that ketone bodies first cause OS and impair mitochondrial function, and later promote the antioxidant defence programme to maintain redox homeostasis [22].

It has been proposed that hypogonadism or low testosterone level may represent a condition of OS [23,24]. The TAC is lower in individuals with a low total testosterone level, and significant strong positive associations between the TAC and sex hormone concentrations have been reported [23]. Although the kinetics of the testosterone response during prolonged fasting in healthy adults remains unknown, research findings indicate that prolonged fasting (>48 h) leads to a reduction in testosterone levels in healthy adults [25,26,27,28] but does not seem to suppress the fasting-induced increase in the TAC [10]. It may be postulated that, in life-threatening situations such as prolonged fasting, energy can be allocated to suppress the reproductive system, which increases survivorship [29,30,31].

Early prevention targeting the fundamental molecular causes of a disease is crucial because it may delay or prevent disease progression. The implementation of a self-empowering, low-cost, efficacious therapeutic approach for health enhancement would be a valuable addition to the management of health. The main aims of this study were to assess the redox status kinetics during a 6-day fasting period and after 1 week of refeeding in non-obese healthy young men and to examine whether the observed changes parallel the changes in ketone and testosterone levels, and the autonomic nervous system. Although the exact pattern of redox kinetics during prolonged fasting is unknown, we hypothesized that the TAC would increase progressively during fasting, that OS would increase only during the first 2–4 days of fasting, and that a longer 6-day fast would cause adaptations to OS, such as a suppressed MDA level. We note that a recent study found that the glucose, ketone, and catecholamine responses to fasting were temporary and occurred only during fasting and not upon resumption of the regular diet following a 6-day fast [19]. Thus, we expected that the change in redox status may also be temporary and would return to the pre-fasting state upon return to the usual eating habits.

## 2. Materials and Methods

### 2.1. Ethical Approval

The present study was approved by The Lithuanian Sports University Bioethics Committee (No. MNL-SFZ(M)-2021-339; 4 February 2021) and was conducted according to the guidelines laid down in the Declaration of Helsinki. Written informed consent was obtained from all participants during the initial visit. The trial was registered at ClinicalTrials.gov (No. NCT05545943; 19 September 2022).

### 2.2. Participants

**Figure 1:**
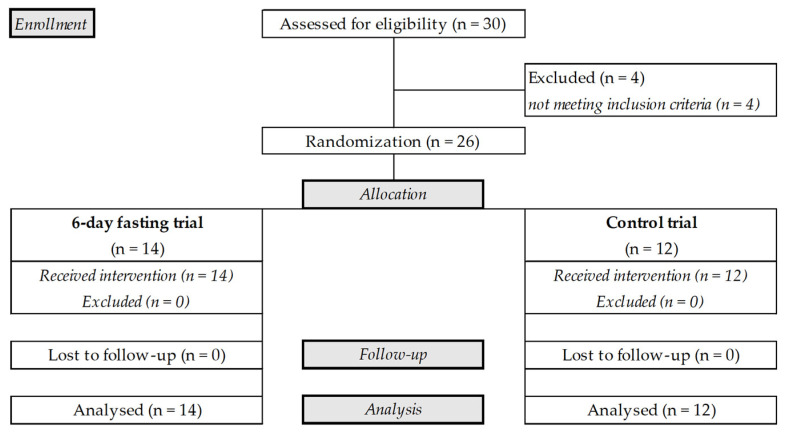
CONSORT flowchart of this study.

Thirty men in total were assessed for eligibility. The eligibility criteria were as follows: (i) male; (ii) no blood or needle phobia; (iii) body mass index (BMI) 18.5–29.9 kg/m^2^; (iv) no history of alcohol, nicotine, or drug abuse; (v) no medications and/or dietary supplements that could affect the experimental variables; and (vi) no history of any eating disorder, oncological, metabolic, cardiovascular, skeletal, neuromuscular, pulmonary disorder or disease, mental disability, or condition that could be worsened by fasting or that could affect the experimental variables. Participants were excluded if they had a history of an eating disorder, were participating in a weight-reduction programme, followed a low-carbohydrate diet, or had been engaging in regular physical activity (>3 times per week and >150 min of moderate-intensity or >75 min of vigorous-intensity activity per week) for >3 months. Four participants did not meet the inclusion criteria and were excluded: one was obese (BMI > 30.0 kg/m^2^), one was a smoker, one was engaged in regular physical activity, and one had a needle phobia. The final sample included 26 men who met the inclusion criteria (Figure 1). Their mean age was 29.4 ± 7.0 years, body mass (BM) was 84.8 ± 14.7 kg, and BMI was 24.9 ± 2.5 kg/m^2^.

### 2.3. Study Design and Experimental Protocol

We conducted a single-centre, parallel-group, single-blind randomized controlled trial at the Institute of Sports Science and Innovations, Lithuanian Sports University, from February 2021 to September 2022. All participants were recruited via public announcements and social networks. One week before the experimental trial, the participants attended a familiarization session during which they were introduced to the study procedures. Participants were asked to refrain from alcohol, caffeine, and antihistamines for at least 72 h before the experimental measurements and strenuous physical activity during the experiment.

The participants arrived after overnight fasting at the laboratory and every experimental trial began at 8:00–9:00 a.m. to control for circadian hormone fluctuations. Upon arriving, anthropometric variables were measured and the Polar H2 heart rate sensor (Kempele, Finland) was attached to the chest. The participant was asked to rest in a semi-recumbent position for 20 min in a quiet room at an ambient temperature of 24 °C with 60% relative humidity. The pulmonary gas and cardiovascular autonomic responses were recorded during the last 10 min of the resting period. Venous samples were obtained for the measurement of testosterone and MDA concentrations, and TAC and capillary blood samples were obtained for the measurement of ketone and glucose concentrations.

**Figure 2:**
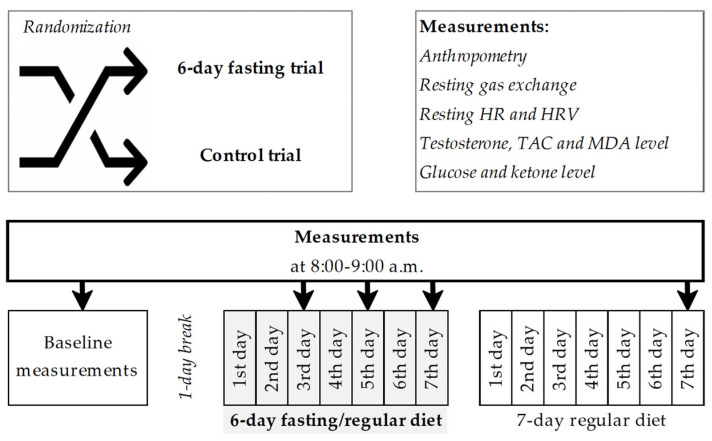
Schematic representation of the protocol. Notes: HR, heart rate; HRV, heart rate variability; TAC, total antioxidant capacity; MDA, malondialdehyde.

Each participant rested after baseline evaluation for 1 day before starting one of the randomly prescribed trials: a 6-day fast (n = 14, age: 30.4 ± 7.0 years, weight: 88.3 ± 13.6 kg, BMI: 25.7 ± 2.4 kg/m^2^) or a control trial (n = 12, age: 28.3 ± 7.2 years, weight: 80.3 ± 15.4 kg, BMI: 23.8 ± 2.3 kg/m^2^). The participants were allocated to the two groups using blocked randomization and a computer-generated list of random numbers that had been prepared by an investigator with no involvement in this study. During the fasting trial, the participants were instructed to follow a prescribed zero-calorie diet, with water provided ad libitum, and the control trial participants were instructed to maintain their previous eating habits over a period of 6 days. On days 2 and 4, and at the end of each trial, the previously described baseline variables were measured again. Participants in both trials then followed their regular diet for 7 days, and all measurements were repeated on day 7 of their regular diet. No participants in the control or experimental trial were lost to follow-up, and the data for all 26 participants were available for the analyses (Figure 1). A schematic representation of the protocol procedures is represented in Figure 2.

### 2.4. Anthropometric Measurements

BM (kg) was measured using a Tanita DC-430U body composition analyser (Tokyo, Japan), and height was measured using a Harpenden anthropometer set (Holtain Ltd., Crymych, Dyfed, UK) while the participants were wearing only underwear and were barefoot. BMI (kg/m^2^) was calculated as BM/height^2^, and body surface area (BSA; m^2^) was calculated as 128.1 × weight^0.44^ × height^0.60^ [32].

### 2.5. Resting Gas Exchange Analysis

Oxygen uptake (*V*O_2_) and carbon dioxide production (*V*CO_2_) were monitored every 5 s on a breath-by-breath basis using a mobile spirometry system (Oxycon Mobile, Jaeger/VIASYS Healthcare, Hochberg, Germany), which was automatically calibrated following the manufacturer’s instructions. Substrate utilization was determined using the respiratory quotient (RQ = *V*CO_2_/*V*O_2_). The RQ values for fat, protein, and carbohydrate were assumed as 0.7, 0.8, and 1.0, respectively [33]. Fat oxidation (FATox, g/min) and total carbohydrate oxidation (CARBox, g/min) were calculated using the following non-protein stoichiometric equations [34]: FATox = [1.695 × (*V*O_2_ × 60)] − [1.701 × (*V*CO_2_ × 60)] and CARBox = [4.585 × (*V*CO_2_ × 60)] − [3.226 × (*V*O_2_ × 60)].

### 2.6. Cardiovascular Autonomic Function

The Polar H2 heart rate (HR) sensor with a chest strap was used to record RR intervals. Each downloaded 10 min RR interval file was analysed using Kubios HR Variability Analysis Software 2.0 (The Biomedical Signal and Medical Imaging Analysis Group, Department of Applied Physics, University of Kuopio, Kuopio, Finland) for analysis of HR and HR variability. The trends were removed from the data by using the smoothness priors method, and the medium level of custom filters was used for artefacts and noise correction. In the time domain, we assessed the mean of the RR intervals or, correspondingly, the mean HR, which reflects the combined modulation of both sympathetic and parasympathetic branches of the autonomic nervous system, and the root mean square of successive differences (RMSSD), which reflects the parasympathetic nervous system activity [35,36,37]. In the frequency domain, after performing a fast Fourier transformation analysis, we assessed the low-frequency (LF) power, which reflects the combined modulation of both sympathetic and parasympathetic branches of the autonomic nervous system, and high-frequency (HF) power, which reflects the parasympathetic nervous system activity [36,37]. A logarithmic transformation (Ln) was performed for time and frequency domains to correct for the skewness of the distributions [38,39].

### 2.7. Measurement of Capillary Blood Ketone and Glucose Concentrations

The capillary blood ketone and glucose concentrations were assessed using an Abbott FreeStyle Optium Neo H blood ketone monitoring system (Doncaster, Australia) using blood obtained from a finger-prick sample.

### 2.8. Measurement of Serum Testosterone Concentrations

Blood samples for the measurement of serum total and free testosterone concentrations were collected directly into a 3.5-mL vacutainer tube containing a silica clot activator and polymer gel for serum separation (Becton Dickinson, Franklin Lakes, NJ, USA). Vacuum tubes containing anticoagulant were gently inverted 8–10 times immediately after blood collection. Serum-separating tubes were allowed to clot for 30–60 min at room temperature, and the tubes were centrifuged at 2000× *g* for 15 min at 4 °C. After centrifugation, the blood samples were transferred into 1.5-mL microtubes (FL Medical, Italy), and the microtubes containing the serum were stored at –80 °C until analysis. Testosterone concentrations were measured using solid-phase enzyme-linked immunosorbent assay (ELISA) kits (Cat. No. RE52151 for total testosterone concentration and Cat. No. DB52181 for free testosterone concentration, IBL International GmbH, Hamburg, Germany) and a Spark multimode microplate reader (Tecan, Grödig, Austria) in duplicates, and the average of the two values was used for the analysis. The intra-assay coefficients of variation (CVs) for total and free testosterone concentrations were 1.85% and 2.41%, and the interassay CVs were 3.86% and 2.71%, respectively. The researcher who analysed the serum testosterone concentrations was blinded to the experimental conditions.

### 2.9. Measurement of Plasma MDA Concentration and TAC

Blood samples for measurement of plasma MDA concentration and the TAC were collected directly into a 3-mL vacutainer tube containing tripotassium ethylenediaminetetraacetic acid as the anticoagulant (EDTA-K3; Becton Dickinson). The tubes were centrifuged at 2000× *g* for 15 min at 4 °C. After centrifugation, blood samples were transferred into 1.5-mL microtubes (FL Medical), and the plasma microtubes were stored at –80 °C until analysis. The MDA concentration was measured using a solid-phase ELISA kit (Cat. No. E1371Hu, Bioassay Technology Laboratory, Shanghai, China). The TAC was measured colorimetrically with an assay kit (Cat. No. E-BC-K271-M, Elabscience Biotechnology Inc., Houston, TX, USA), which is based on the ABTS method, in which antioxidants in the plasma samples inhibit the oxidation of ABTS to its radical cation (ABTS+), leading to a decrease in absorbance at 734 nm. The results were compared against a Trolox standard as the reference antioxidant. All samples were run in duplicate using a Spark multimode microplate reader (Tecan), and the average of the two values was used for the analysis. The intra-assay CVs for MDA and TAC were 7.40% and 6.82%, and the inter-assay CVs were 7.56% and 7.16%, respectively. The plasma TAC/MDA ratio was calculated as an index of oxidative status. The researcher who analysed the plasma MDA concentration and TAC concentrations was blinded to the experimental conditions.

### 2.10. Statistical Analyses

An a priori power analysis was conducted using G*Power version 3.1.9.7 (Düsseldorf, Germany), following the use of the data involving three participants in each group who completed this study. The primary outcomes for this study were TAC and MDA. Given *η_p_*^2^ = 0.315 for TAC and *η_p_*^2^ = 0.579 for MDA, with an α level of 0.05 and a β value of 80%, power analysis indicated that four participants per group (eight in total) would be required. However, this research is part of a larger study published earlier [19,21], and thus included a larger sample size.

Statistical analyses were performed using IBM SPSS Statistics software (version 28.0.1.0 (142); IBM Corp., Armonk, NY, USA). Normality was examined using the Shapiro–Wilk test. Parametric tests were used to analyse data that followed a normal distribution. A two-way mixed-model repeated-measures analysis of variance (ANOVA) followed by the Bonferroni post hoc test was used to examine the effects of intervention (control and fasting trials) and time for the measures obtained at four times (BM, BMI, BSA, HR, HR variability, substrate oxidation, capillary glucose concentration, serum testosterone concentration, and plasma MDA concentration and the TAC). In case of sphericity violation, the Greenhouse–Geisser estimates were used. A dependent *t*-test for paired samples was used to examine the effect of time for two repeated measures, and an independent-sample *t*-test was used to identify differences between trials. Non-parametric tests were used for data that did not follow a normal distribution. The Friedman test followed by the Wilcoxon signed-rank test was used to examine the effect of time on ketone concentration and the TAC/MDA ratio, and the Mann–Whitney *U* test was used to compare trials. Statistical significance was set at *p* < 0.05. The data are presented as the mean and standard deviation. All graphs were created using GraphPad Prism 10 (version 10.0.0.3; GraphPad Software, San Diego, CA, USA).

## 3. Results

### 3.1. Effects of Fasting on Anthropometric Measures

A mixed-design ANOVA revealed a significant effect of time (*p* < 0.001, *η_p_*^2^ > 0.75) and a significant time × intervention interaction (*p* < 0.001, *η_p_*^2^ > 0.75) on anthropometric measures. Subsequent post hoc tests using the Bonferroni correction showed that BM, BMI, and BSA decreased significantly throughout the fasting period (*p* < 0.001). After the recovery period, BM and all anthropometric values increased (paired *t*-test; *p* < 0.001) but remained lower than the pre-fasting values (paired *t*-test; *p* < 0.001) (Table 1).

### 3.2. Effect of Fasting on Capillary Glucose and Ketone Concentrations

**Figure 3:**
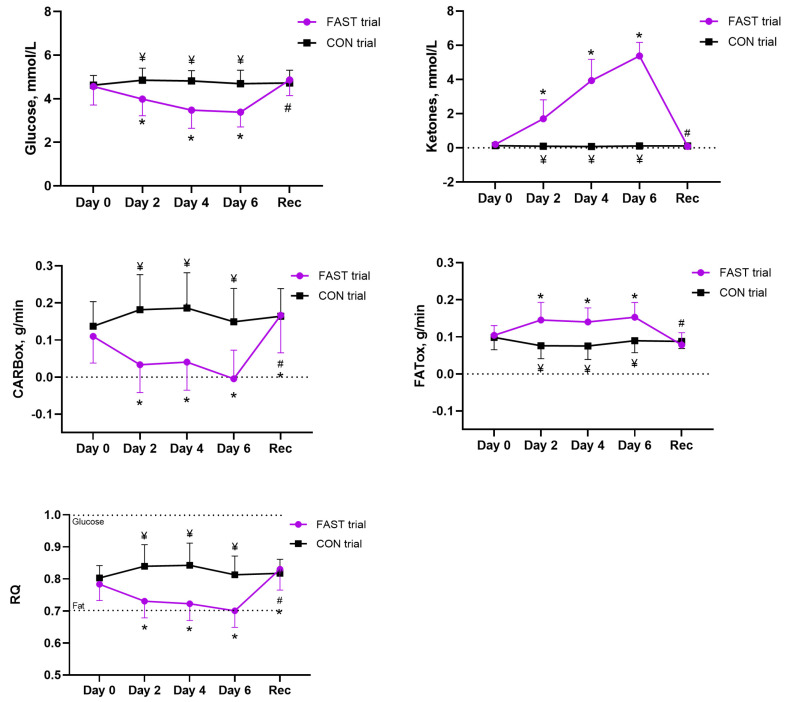
Effect of fasting on capillary concentrations of glucose and ketones, and substrate oxidation. Notes: * *p* < 0.05, compared fasting data with baseline (Day 0); # *p* < 0.05, compared with last day of fast (Day 6); ¥ *p* < 0.05, comparison of both trials. FAST, fasting; CON, control; Rec, recovery; RQ, respiratory quotient, CARBOX, carbohydrate oxidation; FATOX, fat oxidation.

A mixed-design ANOVA revealed a significant effect of time (*p* < 0.001, *η_p_*^2^ = 0.27) and a significant time × intervention interaction (*p* < 0.001, *η_p_*^2^ = 0.33) on capillary glucose concentration. Post hoc tests using the Bonferroni correction showed that glucose concentration decreased significantly throughout the fasting period (*p* < 0.005). During the fasting period, lower glucose concentrations (*p* < 0.003) were observed from day 2 to 6 in the fasting trial than in the control trial. After the recovery period, glucose concentration increased compared with the last day of the fasting period (day 6) (paired *t*-test; *p* < 0.001) and reached the pre-fasting value (paired *t*-test; *p* > 0.05) (Figure 3).

The Friedman test revealed a significant effect of fasting time (*p* < 0.001) on capillary ketone concentration. A subsequent Wilcoxon signed-rank test showed that ketone concentration increased significantly throughout the fasting period (*p* < 0.001). During the fasting period, higher ketone concentrations were observed from days 2 to 6 in the fasting trial than in the control trial (Mann–Whitney *U* test; *p* < 0.003). After the recovery period, ketone concentration decreased compared with the last day of the fasting period (day 6) (Wilcoxon signed-rank test; *p* < 0.001) and reached the pre-fasting value (Wilcoxon signed-rank test; *p* > 0.05) (Figure 3).

### 3.3. Effects of Fasting on Substrate Oxidation

A mixed-design ANOVA revealed a significant effect of time (*p* < 0.030, *η_p_*^2^ = 0.12–0.18) and a significant time × intervention interaction (*p* < 0.001, *η_p_*^2^ = 0.29–0.33) on RQ, CARBox, and FATox (Figure 3). Subsequent post hoc tests using the Bonferroni correction showed that RQ and CARBox decreased significantly and FATox increased throughout the fasting period (*p* < 0.001). During the fasting period, lower RQ (unpaired *t*-test; *p* < 0.001) and CARBox, and higher FATox (unpaired *t*-test; *p* < 0.001) were observed from days 2 to 6 of the fast in the fasting trial than in the control trial. After the recovery period, RQ and CARBox increased and FATox decreased compared with the last day of the fasting period (day 6) (paired *t*-test; *p* < 0.001). RQ and CARBox were higher (paired *t*-test; *p* < 0.030), and FATox lower than the pre-fasting values (paired *t*-test; *p* = 0.005).

### 3.4. Effects of Fasting on HR and HR Variability

A mixed-design ANOVA revealed a significant effect of time on the RR interval (*p* = 0.006, *η_p_*^2^ = 0.16) and HR (*p* = 0.009, *η_p_*^2^ = 0.15) (Table 2). Only a tendency was observed for the time × intervention interaction on the RR interval (*p* = 0.060, *η_p_*^2^ = 0.10) and HR (*p* = 0.133, *η_p_*^2^ = 0.70). Post hoc tests using the Bonferroni correction showed that the RR interval decreased (*p* < 0.007) and HR increased (*p* < 0.005) after days 2 and 4 of fasting, and that both variables reached the pre-fasting values after day 6 of the fast (*p* > 0.05) (Figure 3 and Table 2). No significant effect of time or time × intervention interaction on HR variability was observed (mixed-design ANOVA, *p* > 0.05) (Table 2).

### 3.5. Effect of Fasting on Testosterone Concentration

**Figure 4:**
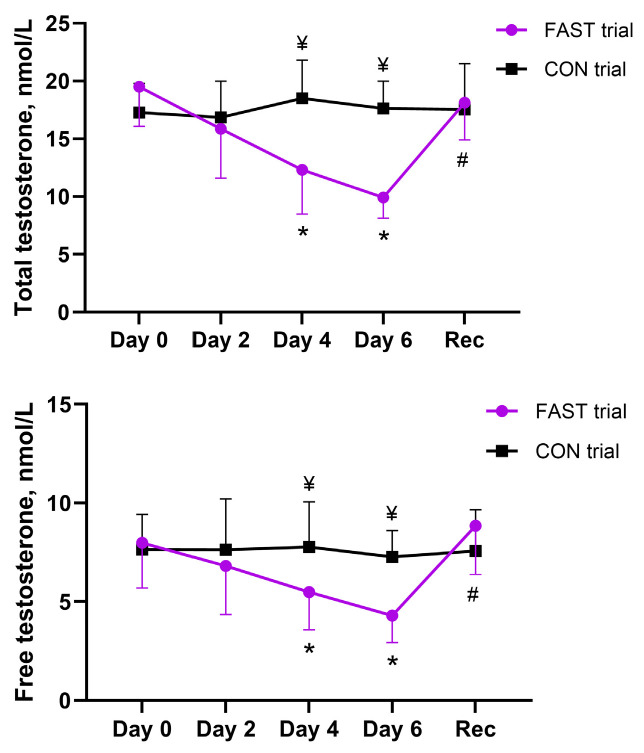
Effect of fasting on serum testosterone concentration. Notes: * *p* < 0.05, compared fasting data with baseline (Day 0); # *p* < 0.05, compared with last day of fast (Day 6); ¥ *p* < 0.05, comparison of both trials. FAST, fasting; CON, control; Rec, recovery.

A mixed-design ANOVA revealed a significant effect of time (*p* < 0.001, *η_p_*^2^ = 0.41 for total testosterone and *η_p_*^2^ = 0.24 for free testosterone concentrations) and a significant time × intervention interaction (*p* < 0.001, *η_p_*^2^ = 0.50 for total testosterone and *η_p_*^2^ = 0.19 for free testosterone concentrations) on serum testosterone concentration (Figure 4). Subsequent post hoc tests using the Bonferroni correction showed that total testosterone concentration decreased significantly after days 2 to 6 of fasting (*p* ≤ 0.003) and free testosterone significantly decreased after days 4 and 6 of the fast (*p* < 0.001). During the fasting period, lower testosterone concentrations (*p* ≤ 0.01) from day 4 to 6 were observed in the fasting trial than in the control trial. After the recovery period, serum testosterone concentration increased compared with the last day of the fasting period (day 6) (paired *t*-test; *p* < 0.001) and reached the pre-fasting value (paired *t*-test; *p* > 0.05).

### 3.6. Effects of Fasting on the Plasma TAC and MDA Concentration

**Figure 5:**
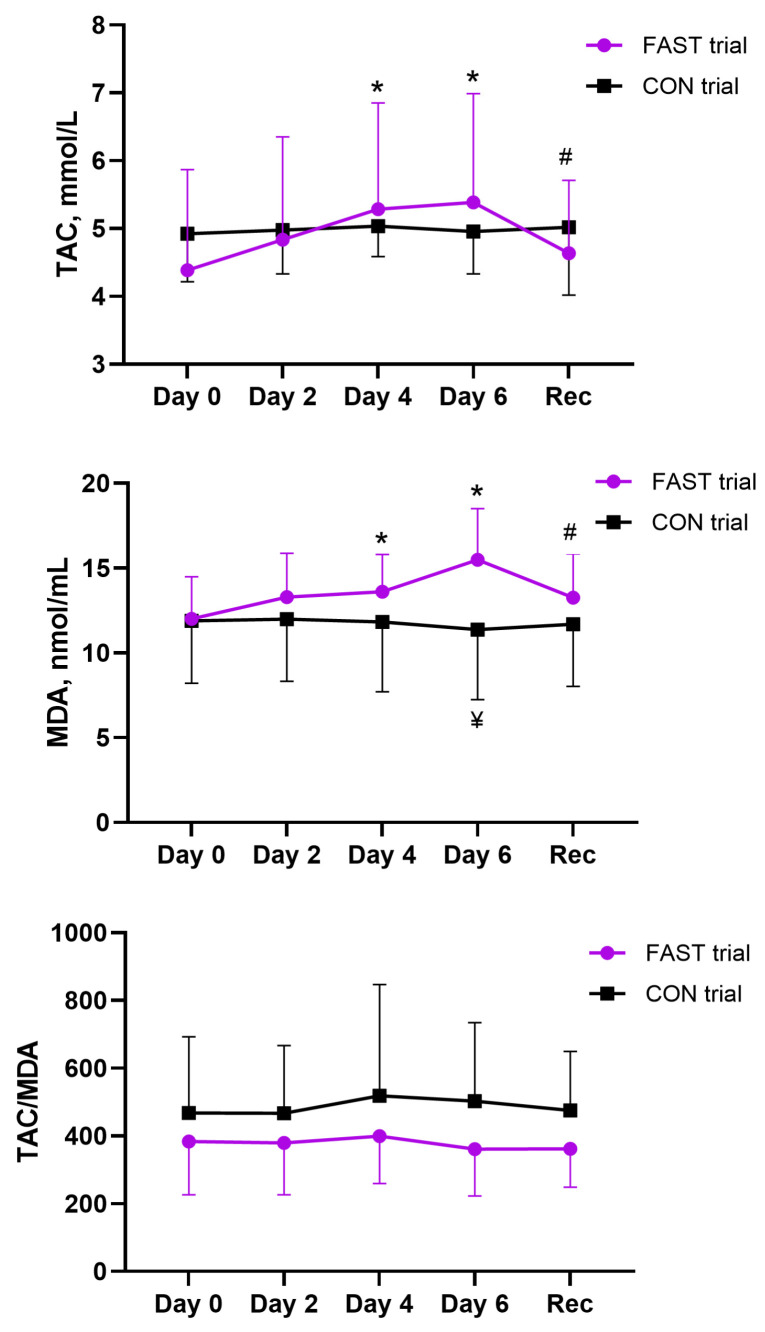
Effect of fasting on total antioxidant capacity and malondialdehyde concentration. Notes: * *p* < 0.05, compared fasting data with baseline (Day 0); # *p* < 0.05, compared with last day of fast (Day 6); ¥ *p* < 0.05, comparison of both trials. FAST, fasting; CON, control; Rec, recovery; TAC, total antioxidant capacity; MDA, malondialdehyde; TAC/MDA, ratio of total antioxidant capacity and malondialdehyde.

A mixed-design ANOVA revealed a significant effect of time (*p* = 0.003, *η_p_*^2^ = 0.18) and a significant time × intervention interaction (*p* = 0.011, *η_p_*^2^ = 0.14) on the plasma TAC (Figure 5). Subsequent post hoc tests using the Bonferroni correction showed that the TAC increased significantly after days 4 and 6 of fasting (*p* ≤ 0.002). The TAC did not differ between the control and fasting trials. After the recovery period, the TAC increased compared with the last day of the fasting period (day 6) (paired *t*-test; *p* = 0.025) and reached the pre-fasting value (paired *t*-test; *p* > 0.05).

A mixed-design ANOVA revealed a significant effect of time (*p* = 0.007, *η_p_*^2^ = 0.18) and a significant time × intervention interaction (*p* < 0.001, *η_p_*^2^ = 0.29) on plasma MDA concentration (Figure 5). Surprisingly, post hoc tests using the Bonferroni correction showed that MDA concentration increased significantly after days 4 and 6 of fasting (*p* ≤ 0.044) and was higher after day 6 in the fasting than in the control trial (*p* = 0.007). After the recovery period, MDA concentration decreased compared with the last day of the fasting period (day 6) (paired *t*-test; *p* = 0.002) and reached the pre-fasting value (paired *t*-test; *p* > 0.05). The Friedman test revealed no significant effect of fasting time (*p* > 0.05) on the TAC/MDA ratio, and this ratio did not differ between the control and fasting trials (Mann–Whitney *U* test; *p* > 0.05) (Figure 5).

## 4. Discussion

Over the past decade, fasting has gained worldwide popularity because of its potential health benefits [8,9,40]. In the present study, we investigated the redox responses to a 6-day fasting intervention in a cohort of healthy young men. Increases in the sympathetic nervous system and ketone responses and decreases in testosterone concentrations were seen after 2 days of fasting. Surprisingly, delayed increases in both the antioxidant capacity and OS were evident after 4 and 6 days of fasting, but the balance of the redox status was maintained. The overall maintained redox balance is crucial for maintaining a healthy status in young adults. These changes were transient and reverted to their pre-fasting state once fasting was discontinued.

Participants’ successful completion of this study demonstrate good tolerability of the 6-day fasting. As expected, we found that prolonged fasting caused a progressive increase in ketone concentration and decrease in glucose concentration. As a result, FATox increased and CARBox decreased. Interestingly, after the participants resumed their usual eating habits, CARBox increased and FATox decreased, changes that may have facilitated a more effective delivery of carbohydrates. Nevertheless, the RQ indicated that, during fasting and after the resumption of the usual eating habits, a predominance of protein oxidation, which ranged from 0.7 to 1.0 [33], was observed. This finding is consistent with the results of a previous study involving 10-day complete fasting [18].

The metabolic shift towards fat oxidation and ketolysis during fasting or a ketogenic diet is associated with mitochondrial stress [41,42]. It has been proposed that the early increase in OS level in mitochondria caused by a ketogenic diet or during periods of food deprivation may activate adaptive (protective) pathways, which increase the production of antioxidants and help to resolve OS [41,42]. Interestingly the increase in MDA concentration was delayed, and the increased OS triggered enhanced antioxidant defence, which meant that the redox status was maintained unchanged through the fasting period. A previous study reported that fasting decreases antioxidant vitamin C levels, which compromises the increase in the TAC [10]. However, the TAC remains elevated and this can be explained by a fasting-induced increase in bilirubin [43] and uric acid concentrations in humans [10]. Both bilirubin and uric acid function act as antioxidants and scavenge radicals [44,45,46,47]. Uric acid contributes about 60% of the scavenging capacity in plasma [44].

Interestingly, contrasting results have been observed during 10 days of near-complete (250 kcal/day) calorie deprivation, in that lipid peroxidation was significantly lower after than before fasting [40,48]. This finding suggests that near-complete calorie deprivation acts protectively by activating solely the antioxidant machinery. By contrast, in the current study, complete calorie deprivation caused both progressive OS (e.g., increased MDA concentration) and antioxidant defence (e.g., increased TAC). The redox status was maintained, as indicated by an unchanged TAC/MDA ratio throughout the fasting period. One possible explanation may be related to differences in the increase in ketone level evoked by calorie deprivation; that is, a larger increase in ketone level induced by complete fasting may have caused greater MDA concentration in the current study. Because the metabolic switch occurs in the fasting state [14], the smaller change in glucose concentration observed in previous studies by Wilhelmi de Toledo et al. [40] may reflect a smaller change in ketone concentration.

It has been proposed that evoked stress can increase OS [20]. Another proposed explanation for the differences in the response of MDA to fasting may reflect differences in the evoked stress response to different extents of calorie deprivation (0 vs. 250 kcal/day) because greater calorie deprivation causes greater activation of the sympathetic–adreno–medullar axis. Consistent with the previously observed sympathetic activation (e.g., increased epinephrine concentration) induced in the early phase of complete fasting [19,21], we observed higher HR and lower RR intervals between days 2 and 4 of fasting compared with the baseline values. Similar to the epinephrine response after a 6-day fast [19], the RR and HR values returned to the pre-fasting levels and remained stable throughout the recovery period in our study. RMSSD and HF power reflect the parasympathetic nervous system activity [35,36,37] but were not affected by fasting in our study. Surprisingly, MDA concentration increased only after 4 and 6 days of fasting and returned to the pre-fasting level after resumption of the normal diet.

The effects of testosterone are energetically expensive and, in life-threatening situations, energy can be allocated to balance reproductive system function and survivorship [29,30,31]. In our study, serum-free and total testosterone concentrations responded to energy imbalance, and prolonged fasting led to a greater decrease in free and total testosterone concentrations. It is well established that short-term fasting decreases testosterone concentration, potentially due to a decrease in luteinizing hormone (LH) concentration during short-term fasting [25,26,27]. Intriguingly, more prolonged fasting (8-days) shows that the decrement in testosterone concentration is not accompanied by changes in LH concentrations [28]. Thus, it appears unlikely that a decrease in LH is the cause of the suppression of reproductive axis activity during more prolonged fasting periods. However, Letkiewicz et al. [28] observed that the reduction in testosterone concentration was accompanied by a decrease in testicular volume. Testicular size has significant effects in andrology; males with smaller testes have lower testosterone concentrations [49]. In aged animal models, it has been established that reduced expression of key enzymatic and non-enzymatic antioxidants in Leydig cells can result in excessive oxidative stress, potentially leading to a decline in testosterone secretion [50]. In the current study, systemic oxidative status (MDA/TAC) was not affected; however, this does not reflect the redox status specific to Leydig cells. Theoretically, suppression of the testosterone level and reproductive system may increase the amount of energy available for energetically costly survival-enhancing functions of immunity [29,30,31] to counteract the MDA-increased inflammatory responses [51].

This current study has limitations. First, the sample size was small, although it was sufficient to detect statistically significant changes in both the TAC and MDA concentration. Second, this study included only healthy young men, and this may limit the direct extrapolation of our conclusions to other demographics, such as women or different age ranges. Third, the causality of the suggested mechanisms could not be established by this study. Future work with larger sample sizes is necessary to confirm any cause–effect relationships.

In conclusion, 6 days of fasting activated mechanisms causing changes in both the antioxidant defence and OS, although the redox balance was maintained. Consistent with this response, ketone concentration and sympathetic nervous system activity increased, and testosterone concentration decreased during fasting. However, all variables returned to their baseline, pre-fasting state after resumption of the usual eating habits. These findings highlight the temporary adaptive nature of the body’s responses to prolonged fasting.

## Figures and Tables

**Table 1:** Effect of fasting on anthropometric measures.

	FAST Trial (n = 14)					CON Trial (n = 12)				
	Day 0	Day 2	Day 4	Day 6	Rec	Day 0	Day 2	Day 4	Day 6	Rec
**BM (kg)**	88.3 (13.6)	86.4 * (13.2)	84.5 * (13.0)	82.9 * (12.7)	86.1 *# (13.4)	80.3 (15.4)	80.6 (15.4)	80.6 (15.4)	79.8 (14.9)	80.4 (15.7)
**BMI (kg/m^2^)**	25.7 (2.42)	25.2 * (2.48)	24.7 * (2.37)	24.2 * (2.34)	25.1 *# (2.35)	23.8 (2.26)	23.9 (2.18)	23.9 (2.21)	23.7 (2.12)	23.9 (2.25)
**BSA (m^2^)**	2.10 (0.20)	2.08 * (0.20)	2.06 * (0.20)	2.05 * (0.19)	2.08 *# (0.20)	2.02 (0.21)	2.02 (0.21)	2.02 (0.21)	2.00 (0.22)	2.00 (0.23)

Notes: * *p* < 0.05, compared with baseline (Day 0); *#* *p* < 0.05, compared with last day of fast (Day 6). FAST, fasting; CON, control; Rec, recovery; BM, body mass; BMI, body mass index; BSA, body surface area.

**Table 2:** Effect of fasting on heart rate and heart rate variability measures.

	FAST Trial (n = 14)					CON Trial (n = 12)				
	Day 0	Day 2	Day 4	Day 6	Rec	Day 0	Day 2	Day 4	Day 6	Rec
**HR (b/min)**	64.9 (11.6)	72.4 * (15.8)	70.0 * (14.0)	67.3 (15.4)	64.1 (12.3)	66.2 (13.6)	68.7 (12.4)	68.7 (10.1)	70.3 (11.2)	69.0 (11.8)
**RR interval (ms)**	953.2 (164.4)	879.7 * (193.4)	870.1 * (157.0)	934.0 (203.0)	955.1 (178.7)	920.0 (151.7)	886.6 (149.0)	887.6 (125.8)	864.7 (119.8)	876.4 (130.5)
**RMSSD (Ln(ms))**	4.05 (0.66)	3.74 (0.84)	3.80 (0.88)	3.91 (0.83)	4.01 (0.81)	4.23 (0.73)	4.12 (0.56)	4.10 (0.56)	3.95 (0.57)	4.11 (0.66)
**LF power (Ln(ms^2^))**	6.98 (0.93)	6.73 (0.80)	6.52 (0.81)	6.52 (0.66)	6.49 (0.81)	7.32 (1.12)	7.30 (0.77)	7.34 (0.75)	7.05 (0.90)	7.08 (0.68)
**HF power (Ln(ms^2^))**	7.20 (1.25)	6.62 (1.45)	6.74 (1.41)	6.83 (1.39)	7.18 (1.36)	7.45 (1.18)	7.29 (1.08)	7.27 (0.95)	6.87 (1.04)	7.14 (1.07)

Notes: * *p* < 0.05, compared with baseline (Day 0). FAST, fasting; CON, control; Rec, recovery; HR, heart rate; RMSSD, root mean square of successive RR interval differences; LF, low frequency; HF, high frequency.

## Data Availability

Public sharing of current data is not possible as participants were not informed of this option and their consent was not obtained.

## Abbreviations

The following abbreviations are used in this manuscript:

ANOVA	Analysis of variance
BM	Body mass
BMI	Body mass index
BSA	Body surface area
CARBox	Carbohydrate oxidation
FATox	Fat oxidation
HF	High-frequency
HR	Heart rate
LF	Low-frequency
Ln	Logarithmic transformation
MDA	Malondialdehyde
OS	Oxidative stress
RMSSD	Root mean square of successive differences
RQ	Respiratory quotient
TAC	Total antioxidant capacity
*V*CO_2_	Carbon dioxide production
*V*O_2_	Oxygen uptake
